# Key factors explaining critical swimming speed in freshwater fish: a review and statistical analysis for Iberian species

**DOI:** 10.1038/s41598-020-75974-x

**Published:** 2020-11-03

**Authors:** Carlos Cano-Barbacil, Johannes Radinger, María Argudo, Francesc Rubio-Gracia, Anna Vila-Gispert, Emili García-Berthou

**Affiliations:** 1 GRECO, Institute of Aquatic Ecology, University of Girona, Maria Aurèlia Capmany 69, 17003 Girona, Spain; 2 Leibniz‐Institute of Freshwater Ecology and Inland Fisheries, Berlin, Germany

**Keywords:** Freshwater ecology, Environmental sciences

## Abstract

Swimming performance is a key feature that mediates fitness and survival in aquatic animals. Dispersal, habitat selection, predator–prey interactions and reproduction are processes that depend on swimming capabilities. Testing the critical swimming speed (*U*_crit_) of fish is the most straightforward method to assess their prolonged swimming performance. We analysed the contribution of several predictor variables (total body length, experimental water temperature, time step interval between velocity increments, species identity, taxonomic affiliation, native status, body shape and form factor) in explaining the variation of *U*_crit_, using linear models and random forests. We compiled in total 204 studies testing *U*_crit_ of 35 inland fishes of the Iberian Peninsula, including 17 alien species that are non-native to that region. We found that body length is largely the most important predictor of *U*_crit_ out of the eight tested variables, followed by family, time step interval and species identity. By contrast, form factor, temperature, body shape and native status were less important. Results showed a generally positive relationship between *U*_crit_ and total body length, but regression slopes varied markedly among families and species. By contrast, linear models did not show significant differences between native and alien species. In conclusion, the present study provides a first comprehensive database of *U*_crit_ in Iberian freshwater fish, which can be thus of considerable interest for habitat management and restoration plans. The resulting data represents a sound foundation to assess fish responses to hydrological alteration (e.g. water flow tolerance and dispersal capacities), or to categorize their habitat preferences.

## Introduction

Swimming performance represents one of the most important features that mediate fitness and survival of fish and other aquatic animals^1–4^. It plays a crucial role in dispersal, migration, habitat selection, predator–prey interactions and reproduction^5–10^. Swimming performance in fish is traditionally assessed using swim tunnels and ecohydraulic flumes^8,11–15^ and can be classified into three categories: sustained, prolonged and burst swimming^16^. Sustained swimming is aerobically fueled and can be maintained for long time periods, typically more than 200 min, without muscular fatigue^17–19^. The maximum swimming speed of which fish are capable is burst swimming, which can be maintained only for shorter periods (typically < 20–30 s) and is fueled anaerobically^16,19^. Prolonged swimming is the transitional mode between sustained and burst swimming and is not barely distinguishable from burst swimming in some species^19^. Prolonged swimming is partly fueled by aerobic and anaerobic metabolism, and can be maintained for intermediate intervals of time (1–200 min)^16,19^.

Since Brett’s work^20^, many authors have opted for determining critical swimming speed (*U*_crit_), as a measurement of prolonged swimming performance, while measuring oxygen consumption rates at the same time^21^. To measure *U*_crit_, individual fish are forced to swim against water flow of increasing velocity until fatigue, i.e. the moment at which the fish can no longer swim and maintain its position in the current^5,22^.

*U*_crit_ is well known to be positively related to body size, including both body length^10,16^ and body mass^23,24^. Swimming performance also depends on body shape^25–28^ and fin form^8,25,29,30^. For example, most of the fast-cruising fish have well streamlined bodies that reduce drag forces and recoil energy losses^31^. Muscle function^32,33^, swimming mode^31,34,35^, and fish behavior^36^ are also important factors that influence fish swimming performance. Thus, *U*_crit_ is strongly size-dependent^36^ and specific to groups of species displaying similar swimming performances^10,14^. *U*_crit_ is also known to depend on the experimental setups and, increases with shorter step-time intervals between velocity increments during the experiment^37^.

Previous studies have shown that abiotic factors, such as water temperature affect the *U*_crit_. In fact, a bell-shaped relationship between temperature and *U*_crit_ has repeatedly been reported^38–40^. This means that *U*_crit_ ascends as temperature rises below the optimum temperature and descends as temperature rises above the optimum temperature^12,21^. Nevertheless, some studies only detected significant decrease in swimming performance with lower water temperatures^12,41^. Similar bell-shaped relationships have also been observed between swimming speed and pH^39^ or salinity^39,42–45^. Other studies have noted the negative effects of several pollutants such as metals and nutrients on fish swimming performance^37,39,46–51^.

The demands of fish on locomotion in flowing water differ from those in still water as fish need to avoid downstream displacement in lotic environments such as rivers and streams^52^. In general, fish species that inhabit in fast flowing riverine habitats tend to show higher *U*_crit_ than those that inhabit in slower flowing riverine or lentic habitats^53,54^. Because of the close relationship between habitat conditions and fish swimming performance, several studies have assessed *U*_crit_ of species in different environments to understand the ecological consequences of anthropogenic perturbations in rivers such as hydrologic alteration, habitat fragmentation^55^, or navigation^10^, and to suggest corresponding mitigation measures. For example, *U*_crit_ has commonly been used to estimate maximum flow velocities in fish passes that assist species to move up or downstream of barriers or that impede the spread of invasive species^14,15,36,56,57^.

The number of studies and the availability of data regarding *U*_crit_ in fish have consistently grown in the last years^14,36^. However, many studies on fish swimming speeds have focused either on salmonids^13^ because of their commercial and recreational interest^42,51,58,59^, and on long-distance migratory fish such as potamodromous and diadromous species^36,60^. By contrast, studies evaluating *U*_crit_ for many other species are rather limited^61^. This is particularly the case for many Mediterranean fish^62^, specifically for rare or local endemic species, which are frequently threatened^63^. Thus, general knowledge on the effects of factors such as body length and temperature on swimming performance in many of these Mediterranean fish species is lacking. Moreover, many regions in the world such as our study area, the Iberian Peninsula, are increasingly invaded by alien species. It has been shown that alien species replace the more flow-adapted native species in hydrologically altered systems^64,65^. However, the mechanisms by which the invasive species have competitive advantage over native species in calm, stagnant waters are still poorly understood. Therefore, a thorough understanding of the swimming capacities of both native and alien species may provide insights into the reasons of this replacement, which can be a result of great importance for the management of water bodies (e.g. habitat assessments of alien and native species, and development of efficient fish passages at physical or velocity barriers for native fish).

The objectives of this study are: (1) to compile the most comprehensive empirical dataset of *U*_crit_ for Iberian freshwater fishes; (2) to compare the role of species identity, taxonomic affiliation, body length, body shape, time step interval between velocity increments and experimental temperature on *U*_crit_, using for the first time the machine learning technique ‘random forests’ (RF), and (3) to test for differences in *U*_crit_ between native and alien species. We hypothesized that larger fish and more streamlined species would show higher *U*_crit_^5^ and that temperature would be one of the main factors that influence *U*_crit_^39^. Particular temperature effects are expected when experimental temperatures are beyond a species’ ecological thermal range. We also hypothesized that alien species would show weaker swimming performance than native fishes because many successful freshwater invaders in the Iberian Peninsula are considered limnophilic, i.e. preferring lentic habitats, compared to the more flow-adapted, often rheophilic native species^64,65^.

## Results

**Figure 1:**
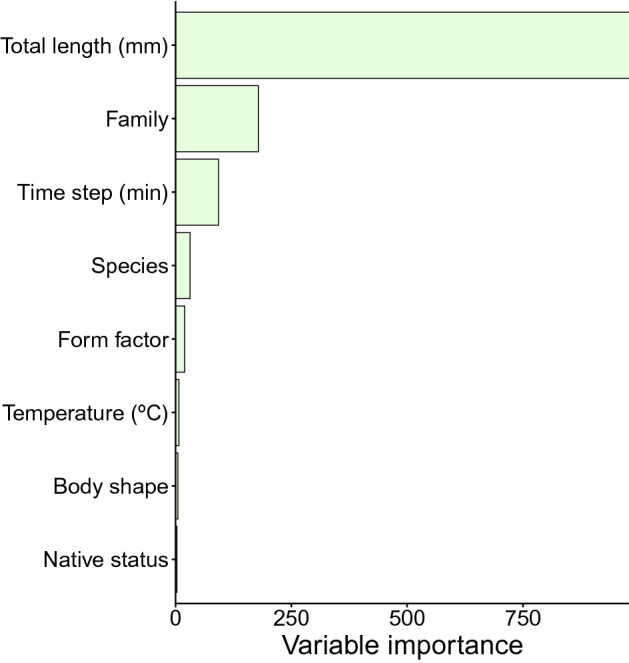
Variable importance of predictors of *U*_crit_ according to the random forest model. Variable importance is the difference in prediction accuracy (i.e. the number of observations classified correctly) before and after permuting a variable, averaged over all trees^123^; and represents the effect of a variable in both main effects and interactions. Total percentage of explained variation was 72.8%. Figure created using R^120^.

**Figure 2:**
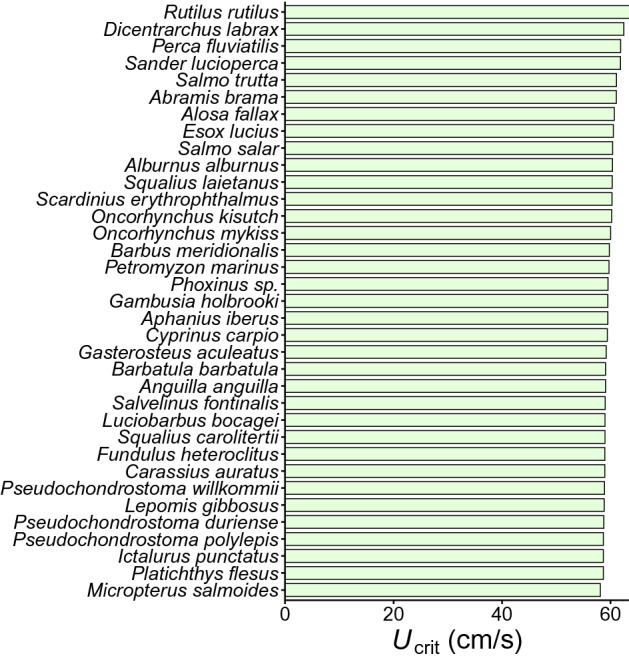
Partial dependence of *U*_crit_ across fish species based on the random forest model. Figure created using R^120^.

The eight explanatory variables used in the RF model (i.e. species identity, family, fish total length [TL], body shape, form factor, time step interval, water temperature and native status) explained 72.8% of the variation in *U*_crit_. The most important explanatory variable out of the eight tested was TL (54.1% variable importance), followed by family (9.9%), time step interval (5.1%) and species identity (1.7%). Form factor (1.1%), temperature (0.4%), body shape (0.3%) and native status (0.2%) were of low importance (Fig. 1). Analysis of partial dependence of *U*_crit_ on TL revealed a steady but nonlinear increase of *U*_crit_ up to a body size TL ≤ 400 mm (Fig. S1) where it reached a plateau, since very few fish in the dataset were longer than 400 mm. In contrast, the partial dependence plot on time step intervals showed a decrease of *U*_crit_ up to a time step interval ≤ 40 min (Fig. S2) where it stabilized. After accounting for fish body length and all other predictor variables, common roach (*Rutilus rutilus*), European bass (*Dicentrarchus labrax*), perch (*Perca fluviatilis*), zander (*Sander lucioperca*) and brown trout (*Salmo trutta*) displayed the highest *U*_crit_. By contrast, largemouth bass (*Micropterus salmoides*), European flounder (*Platichthys flesus*), channel catfish (*Ictalurus punctatus*), Tagus and Douro nase (*Pseudochondrostoma polylepis* and *P. duriense*) and pumpkinseed (*Lepomis gibbosus*) showed the lowest *U*_crit_ (Fig. 2).

**Figure 3:**
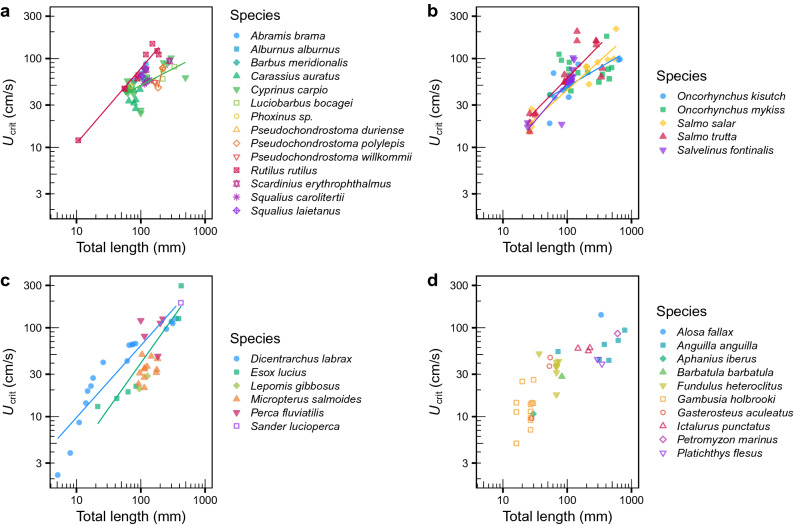
Relationship of *U*_crit_ with fish total length (TL) across species belonging to: (**a**) Cyprinidae and Leuciscidae; (**b**) Salmonidae; (**c**) Percidae, Moronidae, Centrarchidae and Esocidae; and (**d**) other families. Only lines for significant regressions are shown (see Table S1 for statistics). Figure created using R^120^.

**Figure 4:**
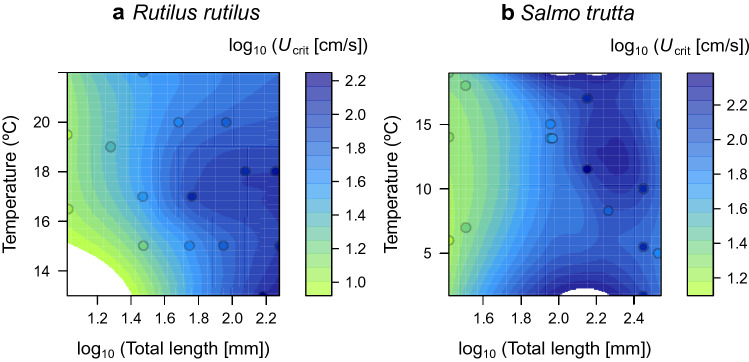
Surface plots relating *U*_crit_ with fish total length (TL) and temperature for two well-studied species: (**a**) *Rutilus rutilus*, and (**b**) *Salmo trutta*. Note log_10_-transformations for *U*_crit_ and TL variables. Figure created using R^120^.

In the analysis of covariance (ANCOVA) model, 84.6% of the variance was explained by the considered explanatory variables: TL, species identity, and their interaction, temperature and time step interval. The TL × species identity interaction was significant, i.e. the slopes of the *U*_crit_—TL relationship varied markedly among species (Table 1, Fig. 3), but was generally positive (approximately linear on a log–log scale) for species with significant relationships. In cyprinids for example, slope was flatter for common carp (*Cyprinus carpio*) than for roach (Fig. 3a, Table S1).The ANCOVA was in agreement with the RF model, showing that fish body length (i.e. log_10_ TL) and fish species identity (and its interaction with length) explained most of the variation in *U*_crit_ (Table 1). In agreement also with the RF model, time step interval showed a significant negative effect on *U*_crit_. Temperature was much less important but significant in the linear model, whereas its quadratic term was not (Table 1). Figure 4 shows the relationship of *U*_crit_ with TL and temperature for two common and well-studied fish species (roach and brown trout). Again, *U*_crit_ showed an increase with fish body length, reaching its maximum at intermediate temperatures, as observed particularly in roach (Fig. 4a).

**Table 1:** Linear model of critical swimming speed (*U*_crit_) in response to total length, fish species, temperature and time step interval. *R*_adj_^2^ = adjusted coefficient of determination in parentheses; d.f. = degrees of freedom; *P* = *P* value.

Response variable (*R*_adj_^2^)	Variable	Sum of squares	d.f.	*P*
log_10_ (*U*_crit_ [cm/s]) (0.846)	log_10_(Total length [mm])	14.474	1	< 0.001
	Species	3.015	34	< 0.001
	Temperature (°C)	0.594	1	< 0.001
	Temperature^2^	0.023	1	0.245
	Time step interval (min)	0.228	1	< 0.001
	log_10_(Total length [mm]) × Species	1.863	25	< 0.001
	Residual	2.403	140

The relationship between *U*_crit_ and TL also varied notably among families, both in intercepts and slopes (Fig. S3, Tables S2 and S3). Cyprinids for example, displayed lower swimming performance than other families studied, especially for longer fish lengths. However, the model accounting for family explained about 1.6% less of the total variance compared to the model with species identity, because there was some variability among species within families, e.g. within cyprinids, leuciscids and salmonids (Fig. 3a,b).

A linear model with only body shape and TL but without species identity explained much less variation, despite being significant (Table S3). The slopes were also significantly different among groups of body shape with fusiform and elongated species showing higher swimming performance for a given length than species with eel-like and short and deep forms (Fig. S4). By contrast, native and alien species did not show significant differences (Fig. S5 and Table S3). The estimated marginal means (EMMs) revealed that (after controlling for length) zander, roach, perch and brown trout were the species with the highest *U*_crit_, whereas European flounder, eel, pumpkinseed and Spanish toothcarp (*Aphanius iberus*) showed the lowest *U*_crit_ (Fig. S6). Comparing the EMMs and the partial dependence of *U*_crit_ on species obtained with the RF model, we observed that both results were highly correlated (*r* = 0.680, Fig. S7) and showed no clear differences (mean difference = -2.19, 95% confidence interval = [-8.74, 4.36], Fig. S8).

Finally, the results of the linear mixed model (LMM) showed that fixed effects (TL, time step interval and water temperature) explained 50.5% of the variation (marginal *R*^2^), whereas the variation explained with the model also including random effects (species) increased up to 88.2% (conditional *R*^2^). This again highlights the differences in *U*_crit_ among species and the heterogeneity of slopes of the *U*_crit_—length relationship. Overall, the LMM revealed a positive effect of TL (coef. = 0.604, SE = 0.072, *P* < 0.001, Fig. S9) and a negative effect of time step interval (coef. = -0.003, SE = 0.002, *P* = 0.004) on *U*_crit_. By contrast, the effect of temperature was statistically not clear in the LMM (coef. = 0.012, SE = 0.002, *P* = 0.987).

## Discussion

This study is the first that comprehensively compiles and investigates a well-established measurement of prolonged swimming performance, i.e. critical swimming speed (*U*_crit_), for 35 freshwater fish species currently inhabiting the Iberian Peninsula. Our results reinforce the importance of several factors that influence *U*_crit_, with fish body length and taxonomic family being the most important predictors, followed by time step interval, species, the form factor, water temperature, species’ body shape and native status.

Analogously to previous studies, our results revealed that fish body length is a key biological factor to understand swimming performance^9,14,16,36^. It is well known that absolute critical swimming speed (*U*_crit_ expressed in cm s^−1^) scales with fish body length^66^, as already described in earlier studies of sustained and prolonged swimming^67^. Furthermore, for many species *U*_crit_ generally increases with the square root of fish length^14,36^. In our study, *U*_crit_ scales with fish body length following the typical allometric equation or power function, which is generally estimated through linear regression of log-transformed variables:

$$ \log U = a + b\cdot\log L $$

where *U* is swimming speed and *L* is fish body length^16^. However, other studies also described the relationship with simple linear regressions without log-transformations^66,68^. Besides body length, it is important to note that body mass may also be an important predictor of *U*_crit_, especially when it comes to comparing swimming abilities among species with different body shapes, swimming and propulsion types^8,16,69,70^. Although not tested in this study, body mass is directly related to body volume and, therefore, to energy expenditure needed to move against the flow^23,71^. Moreover, energy costs of swimming (i.e. the amount of energy necessary to transport one unit of body mass per unit of distance) are negatively associated with body mass because of the lower surface area to volume ratio in larger fish^72,73^. Thus, the surface in contact with water per unit of volume is larger in small fish, increasing the friction drag and the relative dissipated energy^31^. In addition, there is a direct association between body volume and muscle mass and number of myofilaments, which favors swimming performance^21^. As expected, body shape significantly influenced fish swimming performance. Earlier studies showed that body shape also influences the energetic costs associated with swimming^24,69,70^. In general, streamlined fish tend to maximize thrust while minimizing drag and recoil energy losses^72,74^. Correspondingly, fish evolve body forms that enhance steady swimming (i.e. swimming at constant-speed in a straight line) in open-water habitats, high-flow environments, and areas with relatively high competition for patchily-distributed resources^74^. Steady swimming is generally enhanced with a streamlined body shape, a shallow caudal region and a high aspect ratio of the caudal fin^75,76^. In agreement with this, our results showed that elongated and fusiform body shapes are better adapted to swim steadily. On the other hand, species that present the opposite suite of morphological traits such as eel-like and short and deep bodies tend to optimize unsteady swimming (i.e. more complicated locomotor patterns in which changes in velocity or direction occur, such as fast-starts, rapid turns, braking, and burst-and-coast swimming)^28^.

Despite the general *U*_crit_ – body length relationship, we found large variability among fish species as indicated by the significant interaction of TL and species identity and associated contrasting slopes. For example, we found that eastern mosquitofish has lower *U*_crit_ than many other species for a given length, as shown in a previous study^23^. These differences might be due to fish species and populations having evolved over long-term periods, thereby adopting different abilities and strategies towards environmental and ecological conditions^36^. For example, a previous study showed that cyprinids living in fast flowing habitats showed higher *U*_crit_ values compared to fish species preferring slow flowing waters, independently of phylogenetic relationships^77^. Other studies found that long-distance migratory fish show higher swimming capabilities than those migrating over shorter distances^1^. Moreover, other species adapted to a specific environment such as bottom-dwelling or flatfish species, usually perform poorly in *U*_crit_^1,78,79^. Consistent with these earlier findings, our results also indicated that benthic and flatfish species like European flounder have relatively lower *U*_crit_. In addition, our results revealed taxonomic family as a good predictor of *U*_crit_ despite marked differences in lifestyle and form among species within the same family^80^. For example, in salmonids we revealed brown trout as a species with a high estimated *U*_crit_, while brook charr (*Salvelinus fontinalis*) showed comparably lower *U*_crit_ for a given body length. These differences in swimming capacity might be related to differences in their habitat preferences with brook charr being generally found in slow-flowing pools whereas brown trout prefers faster riffle areas^59^. The relationships of habitat preferences and swimming capacity have also been shown in cyprinids and leuciscids because of their distribution in a wide variety of habitats and their associated morphological diversity^77,80,81^. However, we acknowledge that our analyses might have been affected by differences in data availability which might also affect the predictive power of the variable ‘species’.

In agreement with previous research, the duration of the step-test interval had an effect on mean critical velocity^37^. *U*_crit_ increases for short time steps and reaches an asymptote at time step intervals between 30 and 60 minutes^37^, as we observed in our results. Thus, given these differences in swimming performance when using different time intervals, we therefore strongly recommend standardizing and carefully choosing the *U*_crit_ protocol to prevent misleading understanding of fish swimming performances. In addition, we also examined the effects of temperature on *U*_crit_, which is also one of the most important abiotic factors influencing fish swimming performance^72,82–84^. Specifically, the relationship between *U*_crit_ and temperature is commonly described by a bell-shaped curve^39^. Others demonstrated this bell-shaped relationship for juvenile sea bass, whose swimming speed increased as temperature rose from 15 to 25 °C and then decreased^38^. Mechanistically, this can be explained by a general decline of all physiological processes at low temperatures (e.g. a decrease in power generated by the muscle) that also reduces *U*_crit_^13,39^. As temperature increases, there is a positive effect on muscle functioning, and its associated power generation contributes to an increase in swimming performance^85,86^. Nevertheless, when temperature exceeds the optimum range, the oxygen-carrying capacity of the blood decreases and restrains oxygen delivery to the tissues^39^. In contrast to this bell-shaped relationship of temperature and *U*_crit_, we only found a positive relationship with temperature. This lack of observed decline at high temperatures might be due to different reasons. On one hand, the bell-shaped curve is strongly influenced by rates of temperature acclimation previous to the experiment, being most marked in fishes that are exposed to intense temperature change, and increasingly less pronounced with acclimation time^13^. On the other hand, most experiments were conducted only at temperatures that are within the normal thermal tolerance range of a species, i.e. optimum or colder temperatures, rather than covering a long temperature gradient^87^. Finally, some studies revealed asymmetric relationships between temperature and *U*_crit_ showing only significant swimming performance decreases at low temperatures^12,41^.

Our results revealed that both native and alien species have similar prolonged swimming performance, after accounting for body size. This finding is fairly surprising according to the apparent differences in habitat preferences between the two class of groups. Several studies showed that alien fish dominate Iberian reservoir habitats with their artificially stable limnological conditions^88–93^. By contrast, native species, mostly cypriniforms, are considered more adapted to lotic habitats with naturally more fluctuating flow regimes and, in particular, with frequent occurrence of high-flow events^23,94–96^. However, several invasive species that are often classified as limnophilic^97^ showed relatively high swimming capacities (e.g. zander, northern pike [*Esox lucius*], common bleak [*Alburnus alburnus*]). These species have in common that are pelagic and some of them also have high trophic level lifestyles, which has been shown to favor swimming performance and maximum aerobic capacity^80^. It suggests, therefore, that the classification of fish according to their habitat preferences is not always a good proxy of their prolonged swimming performance, and that *U*_crit_ may be more related to ecological demands of species^77^. In addition, not all invasive fish of the Iberian Peninsula are inhabiting reservoirs or lentic habitats, as in the case of the non-native rainbow trout (*Oncorhynchus mykiss*) that rather prefer rivers with moderate to rapid flows^98^. Moreover, the native freshwater fish fauna of the Iberian Peninsula is characterized by a low number of families, but with a considerable degree of diversification of species^98^. Thus, it may be further hypothesized that the high diversity of species and forms could have counteracted the variation in *U*_crit_ across species, independently of their origin (native or invasive). Ultimately, some invaders can colonize novel environments using other swimming strategies, like fast-start swimming^99^, and specific meso- or microhabitats. Thus, *U*_crit_ might not be necessarily the main character determining ecological success and, therefore, invasiveness.

Our results might be limited by different issues. First, reported measurements of *U*_crit_ might be skewed by experimental setups, such as the effects of chamber type and length. Indeed, it has been shown that fish can reach higher *U*_crit_ values in longer flumes^61,100–102^. However, when taking data from different sources, we were not able to control for the effect of the flume characteristics on swimming performance measurements because they are often not reported. Second, small sample sizes and the narrow ranges of investigated fish lengths studied for some species might contribute to the large variability in regression line slopes found in this study. This was considered in our results and only provided significant regression lines (e.g. for species with larger sample sizes). Moreover, issues of data availability also affected the selection of predictors. For example, we were not able to analyze the effect of some variables like fish weight, which is often not provided in swimming performance studies, or habitat preference which is often rather unclear for the endemic species of the Iberian Peninsula^97^.

To sum up, this study showed that fish body length is the most relevant explanatory variable of *U*_crit_ out of the eight considered predictor variables. Other important predictors were fish taxonomic affiliation (family and species identity) and the time step interval between velocity increments used during the experiment. Even though we found overall effects of body shape, form and water temperature on *U*_crit_, their relative importance as predictors were much lower. In contrast to our expectations, we did not find clear differences in *U*_crit_ between native and alien fish species, after accounting for size. Therefore, this suggests that prolonged swimming performance might not be always related to the invasiveness of species in recipient ecosystems, although this needs further testing. We conclude that, besides advancing the fundamental understanding of prolonged swimming performance in Iberian freshwater fishes, our findings also provide the foundation to support their management. The compiled dataset comprises the so far most comprehensive information on *U*_crit_ of the Iberian ichtyofauna. However, we note that swimming speed determined for fishes confined in a respirometer do not necessarily translate directly to free-swimming individuals in the field^101^ and thus should be used cautiously. However, until additional research is conducted on free-swimming fish, *U*_crit_ data represent the best information available^103^. Thus, our results may be used as species-specific estimates of *U*_crit_: (i) to design fish bypasses estimating maximum allowable water velocities in order to improve river connectivity^103^, (ii) to develop barriers for the exclusion of invasive species^14^, (iii) to assess the effects of damming and hydrologic alteration on river fish, and (iv) to categorize fish habitat preferences and restrictions, since a species swimming performance might be a limiting factor of its presence in a given habitat.

## Methods

### Data compilation

We attempted to compile *U*_crit_ data for all the current inland fish species inhabiting the Iberian Peninsula, including native and established alien species. The list of species mainly followed Doadrio et al*.*^104^ and Kottelat & Freyhof^105^ and was completed with few more recently described native species^106^ and alien species lately recorded^107–112^. Out of the 68 native and 32 alien naturalized inland fishes of the Iberian Peninsula, we found *U*_crit_ data for 35 species (18 native and 17 alien), from 79 literature sources published from 1959 to 2020 (Table S4), which include data for 8 species (3 native and 5 alien) from our previous work^23,24,70^. Data extraction occasionally implied digitizing figures, using ImageJ2 software^113^, to estimate *U*_crit_ values that were not provided in tables or within the text of the respective literature. We excluded works that investigated gradients or extreme values beyond the salinity or pH natural range of species, or that investigated the effect of pollution on swimming performance. In addition to *U*_crit_ values, we collated eight additional explanatory variables for further analyses. Besides species identity, family and native status (native *vs*. alien), these included fish body length, body shape, body form factor, time step interval and water temperature as described for the experiments. We used body length rather than body mass due to better data availability. *U*_crit_ and fish body length were converted to uniform units: relative *U*_crit_ (BL s^−1^) was converted to absolute *U*_crit_ (cm s^−1^); fork length (FL) or standard length (SL) were converted to total length (TL) using published length-length relationships^114,115^. We obtained the species-specific body shapes indicating whether a fish has a fusiform (i.e. spindle-shaped and streamlined body), elongated (i.e. tubular body), short and deep (i.e. almost circular and laterally compressed body), or eel-like form (i.e. long and snake-like body) from FishBase^114^. Finally, we calculated the species-specific body form factor (*a*_3.0_)^75^ using the parameters *a* and *b* of the weight-length relationship retrieved from FishBase using the following equation:

$$ a_{3.0} = 10^{{{\log}a - S\left( {b - 3} \right)}} $$

where *S* is the slope of the regression of log *a vs*. *b*. For cases of insufficient data on weight–length relationships to estimate *S*, we used the recommended mean value of −1.358^75^. The form factor is an estimate of the coefficient *a* if exponent *b* was 3. This form factor is commonly used to compare body shape differences among populations or species^116,117^ and increases from eel-like to elongated, fusiform and short and deep body shapes^75^. All the experiments considered were carried out at temperatures within a natural thermal range of each species. Raw data compiled are available at figshare (https://doi.org/10.6084/m9.figshare.10260722).

### Statistical analysis

We used random forest (RF)^118^, as implemented in the package “party”^119^ of the R software^120^, to analyze which of the six predictors best explained *U*_crit_. RF is a machine-learning technique that is frequently used because of their advantages, including computational efficiency on large databases with many correlated predictors, the provision of estimates of variable importance, the ability to impute missing data while maintaining accuracy, and the handling of non-linearities and interactions^118,121^. Specifically, RF computed with package “party” has the advantage of providing unbiased variable selection compared to other software packages, because it is more accurate when predictors are correlated and vary in their measurement scale or number of categories^122,123^. We used species identity, family, native status (native *vs*. introduced) and body shape (eel-like, elongated, fusiform or short and deep) as categorical factors, and TL, time step interval, water temperature and form factor as continuous predictors. In a first step, we searched for the optimal hyperparameters, i.e. number of trees (“ntree”) and number of variables per level (“mtry”) using the “mlr” R-package^124^. Consequently, we used 550 trees to build the RF as increasing the number of trees did not substantially affect the results of explained variation or variable importance^125^, and seven variables were randomly sampled as candidates at each split. We measured the percentage of variation explained (i.e. pseudo-*R*^2^) of the final model obtained. We used the conditional permutation scheme to estimate variable importance, which reflects the true impact of each predictor more reliably than a marginal approach^123^. For species, TL and time step interval, we generated partial dependence plots^126^ to graphically illustrate the conditional effect of a predictor while accounting for other predictors.

We used analysis of covariance (ANCOVA) to further investigate the effects and explanatory power of the predictors considered in the RF and to test for specific hypotheses. The general model included fish TL, species identity, and their interaction to test the assumption of homogeneous slopes in the standard ANCOVA^127^; time step interval and temperature and its quadratic term as predictors, since bell-shaped relationship is commonly accepted as the typical effect of temperature on *U*_crit_^38,39^. Another model included fish TL, species identity, time step interval, temperature and its quadratic term as predictors without considering interaction terms. Similarly to the RF-approach, we used the ANCOVA model to compute estimated marginal means (EMMs), using the “emmeans” package^128,129^, for the species identity factor and to compare the predicted *U*_crit_ values with those obtained using RF. For that purpose, we applied the Bland–Altman analysis^130^, an established protocol for assessing agreement between two different measuring methods, using the “blandr” R package^131^. Specific hypotheses that we tested using ANCOVA were: whether there is an overall difference in *U*_crit_ between (i) native and alien species, (ii) among families, and (iii) among body shape categories, after accounting for fish body length. We did not consider the length × factor interaction when it was clearly non-significant (*P* > 0.10) and thus used a standard ANCOVA in these cases^127^. In all models, TL and the response variable (*U*_crit_) were log_10_-transformed to satisfy the assumptions of normality, homoscedasticity and linearity.

Finally, we used a linear mixed model (LMM) accounting for species-specific differences using the R-package “lme4”^132^ to quantify the relative roles of species and other predictors and further test for heterogeneous slopes. We used *U*_crit_ as response variable, TL, time step interval and temperature as fixed-effect covariates and species as random effects in a random slopes model. This approach allows each species to have different slopes, i.e. the covariates have different effects for each species. The random slopes model was selected over the random intercepts model due to lower AIC values (AIC = -147.3 and AIC = -124.6, respectively) and a significant likelihood ratio test (*χ*^2^ = 52.7, df = 15, *P* < 0.001). We also used the “ranova” function of the R-package “lmerTest”^133^ to test the random-effect terms in the model. Finally, we calculated *p* values and the marginal and conditional *R*^2^^134–137^ with the “lmerTest”^133^ and “MuMIn” R-packages^134–137^, respectively. The marginal *R*^2^ describes the variability explained by the fixed effects, while the conditional *R*^2^ describes the variability jointly explained by the fixed and the random effects.

## Supplementary information

Supplementary Information

## Data Availability

Critical swimming speed data underlying the analyses of this study are available as *.csv files via the figshare repository (https://doi.org/10.6084/m9.figshare.10260722).
