# Supplementary material for: Key factors explaining critical swimming speed in freshwater fish: a review and statistical analysis for Iberian species
Source: Sci Rep. 2020 Nov 3;10:18947. doi: 10.1038/s41598-020-75974-x (PMC7609642; doi:10.1038/s41598-020-75974-x)
Supplement: Supplementary file 1 — Supplementary Information [file 41598_2020_75974_MOESM1_ESM.pdf]

Supporting information

KEY FACTORS EXPLAINING CRITICAL SWIMMING SPEED IN FRESHWATER FISH: A REVIEW AND STATISTICAL ANALYSIS USING IBERIAN SPECIES

Carlos Cano-Barbacil[1*], Johannes Radinger[1,2], María Argudo[1], Francesc Rubio-Gracia[1], Anna Vila-Gispert[1] and Emili García-Berthou[1]

[1] GRECO, Institute of Aquatic Ecology, University of Girona, Girona, Spain

[2] Leibniz-Institute of Freshwater Ecology and Inland Fisheries, Berlin, Germany

*Corresponding author postal address: GRECO, Institute of Aquatic Ecology, University of Girona, Maria Aurèlia Capmany 69, 17003 Girona, Spain

*Corresponding author email address: carlos.cano@udg.edu

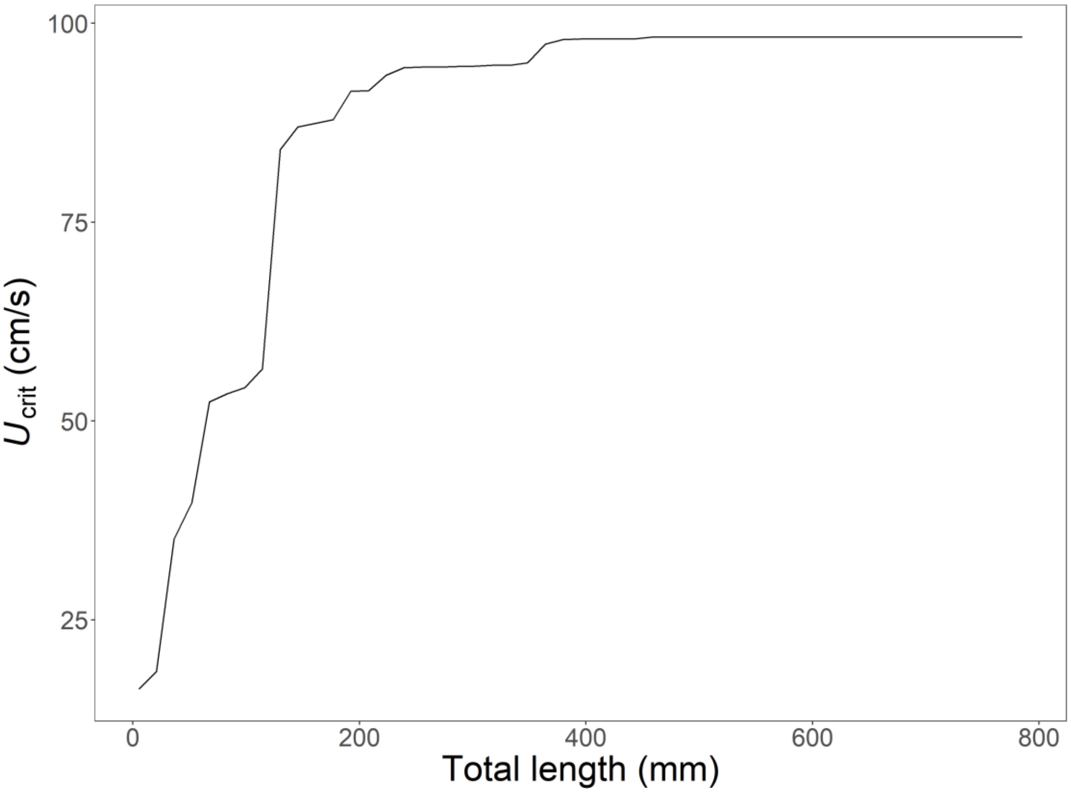

Fig. S1. Partial dependence of U_{crit} on fish total length based on the random forest analysis of Figure 1 (see main text for further information).

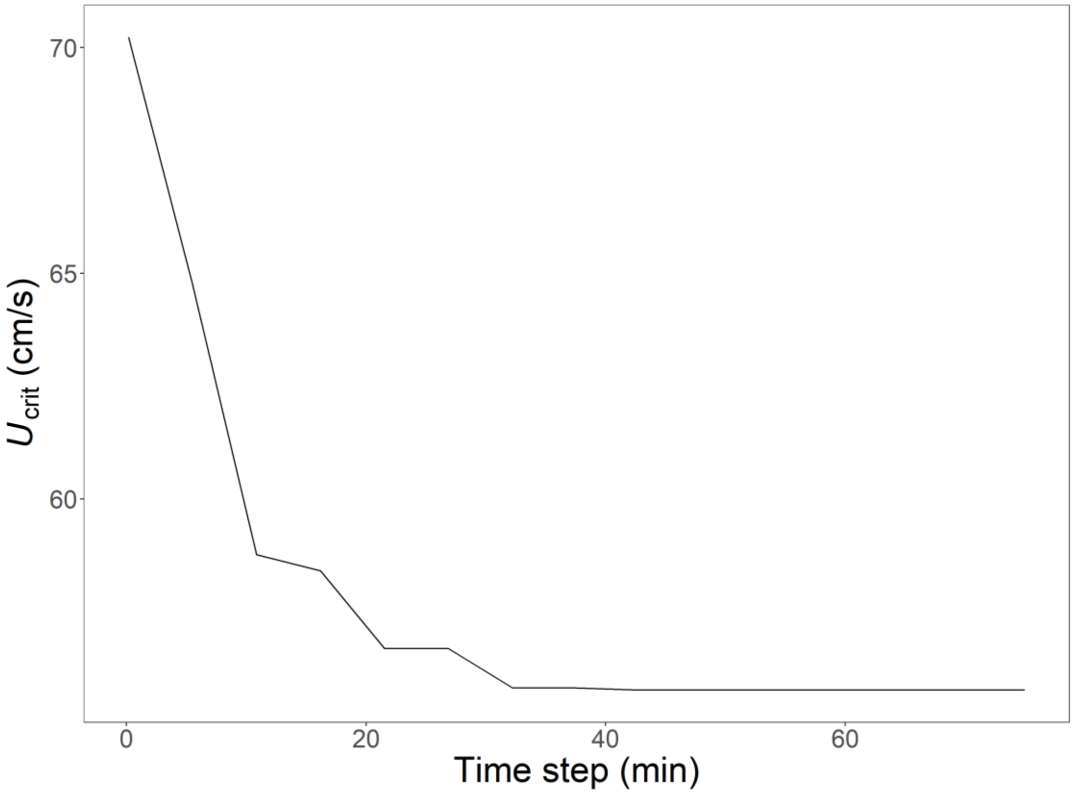

Fig. S2. Partial dependence of U_{crit} on experimental time step based on the random forest analysis of Figure 1 (see main text for further information).

Table S1. Significant linear regression functions of U_{crit} with fish total length (TL) ($\log_{10} U_{crit} = a + b \log_{10} TL$) by species (see also Fig. 3). r^2 = coefficient of determination, P = P value, n = sample size.

Species	*a*	*b*	r^2	*P*	*n*
Cyprinus carpio	0.470	0.929	0.348	0.026	14
Dicentrarchus labrax	0.039	1.062	0.860	<0.001	17
Esox lucius	0.576	0.888	0.905	0.003	6
Oncorhynchus kisutch	-0.224	1.339	0.568	0.007	11
Pseudochondrostoma polylepis	1.719	0.329	0.998	0.001	4
Rutilus rutilus	-0.142	1.133	0.956	<0.001	8
Salmo salar	-0.340	1.424	0.926	<0.001	12
Salmo trutta	0.243	0.986	0.733	<0.001	15
Salvelinus fontinalis	0.572	0.821	0.700	<0.001	14

Table S2. Significant linear regression functions of U_{crit} with fish total length (TL) ($\log_{10} U_{crit} = a + b \log_{10} TL$) by family (see also Fig. S3). r^2 = coefficient of determination, P = P value, n = sample size.

Family	*a*	*b*	r^2	*P*	*n*
Cyprinidae	0.262	1.050	0.437	<0.001	28
Esocidae	0.576	0.888	0.905	0.003	6
Leuciscidae	0.104	1.090	0.623	<0.001	26
Moronidae	0.039	1.062	0.860	<0.001	17
Salmonidae	0.156	1.093	0.624	<0.001	69

Table S3. Selected linear models of U_{crit} (cm/s) with total length (TL, mm) and different predictors. R^2_{adj} = adjusted coefficient of determination in parentheses, d.f. = degrees of freedom, *P* = *P* value, TS = Time step, T = Temperature (°C).

**Selected model (R^2_{adj}, AIC)**	**Variable**	**Sum of squares**	**d.f.**	***P***
$\log_{10}(U_{crit})$~ $\log_{10}$(TL) × Species + T + T^2 + TS (R^2_{adj} = 0.846, AIC = -197.1)	$\log_{10}$(TL)	14.474	1	<0.001
	Species	3.015	34	<0.001
	Temperature	0.594	1	<0.001
	Temperature^2	0.023	1	0.245
	Time step	0.228	1	<0.001
	$\log_{10}$(TL) × species	1.863	25	<0.001
	Residual	2.403	140	
$\log_{10}(U_{crit})$~ $\log_{10}$(TL) × Species (R^2_{adj} = 0.778, AIC = -124.7)	$\log_{10}$(TL)	14.474	1	<0.001
	Species	3.015	34	<0.001
	$\log_{10}$(TL) × species	1.582	25	<0.001
	Residual	3.530	143	
$\log_{10}(U_{crit})$~ $\log_{10}$(TL) + Species + T + T^2 + TS (R^2_{adj} = 0.768, AIC = -130.0)	$\log_{10}$(TL)	14.474	1	<0.001
	Species	3.015	34	<0.001
	Temperature	0.594	1	<0.001
	Temperature^2	0.023	1	0.397
	Time step	0.228	1	0.003
	Residual	4.266	165	
$\log_{10}(U_{crit})$~ $\log_{10}$(TL) × Family (R^2_{adj} = 0.762, AIC = -131.9)	$\log_{10}$(TL)	14.474	1	<0.001
	Family	2.573	16	<0.001
	$\log_{10}$(TL) × Family	0.937	12	<0.001
	Residual	4.618	174	
$\log_{10}(U_{crit})$~ $\log_{10}$(TL) + Body shape (R^2_{adj} = 0.678, AIC = -96.9)	$\log_{10}$(TL)	14.474	1	<0.001
	Body shape	0.994	3	<0.001
	$\log_{10}$(TL) × Body shape	0.333	3	0.025
	Residual	6.801	196	
$\log_{10}(U_{crit})$~ $\log_{10}$(TL) + Native status (R^2_{adj} = 0.637, AIC = -70.6)	$\log_{10}$(TL)	14.474	1	<0.001
	Native status	0.002	1	0.823
	Residual	8.125	201	

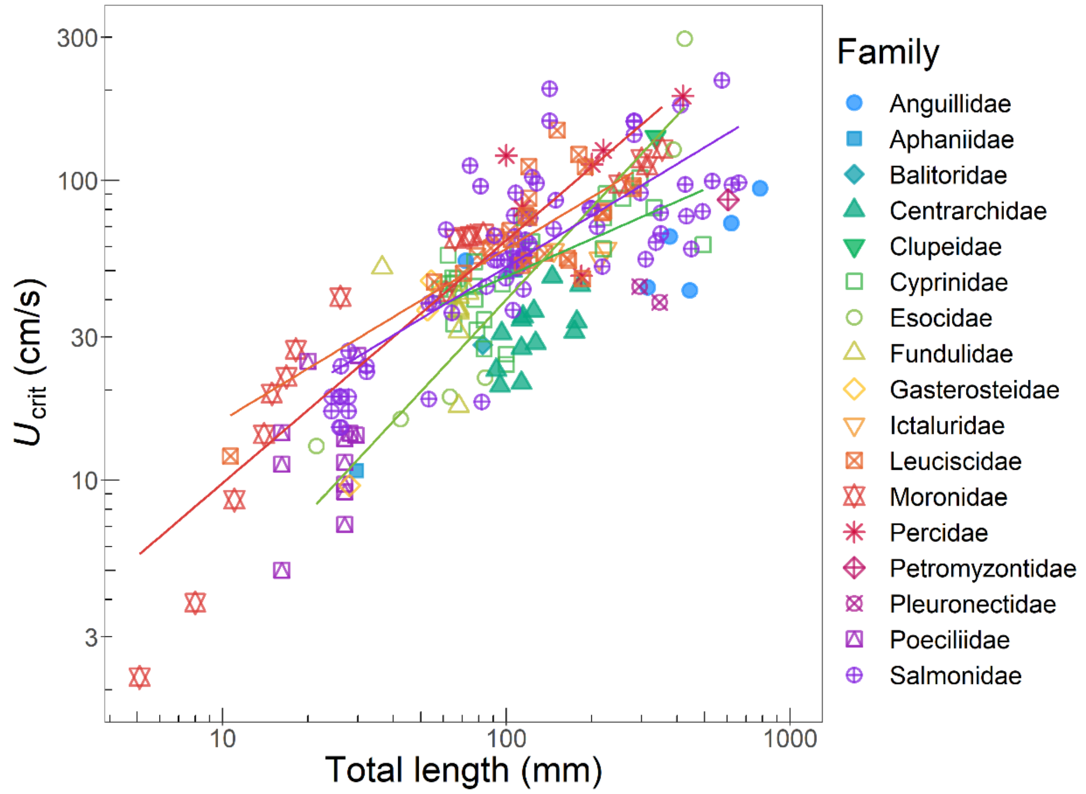

Fig. S3. Relationship of U_{crit} with fish total length (TL) (note log scales) by taxonomic family.

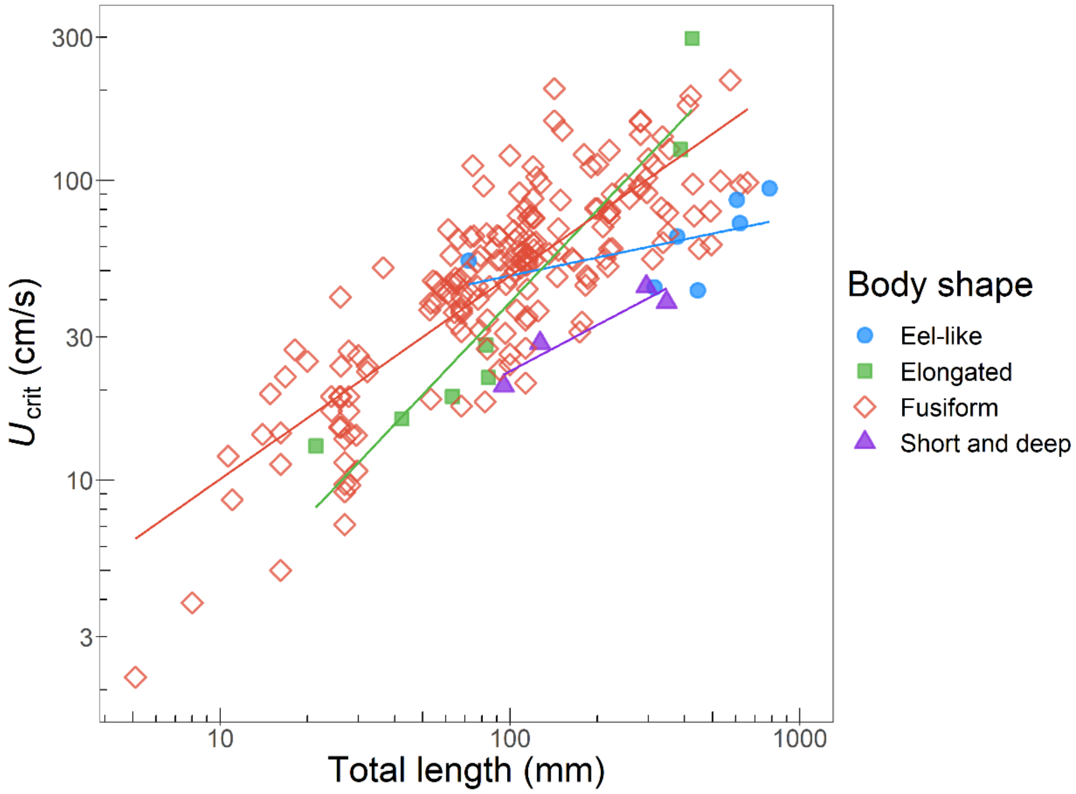

Fig. S4. Relationship of U_{crit} with fish total length (TL) (note log scales) by body shape.

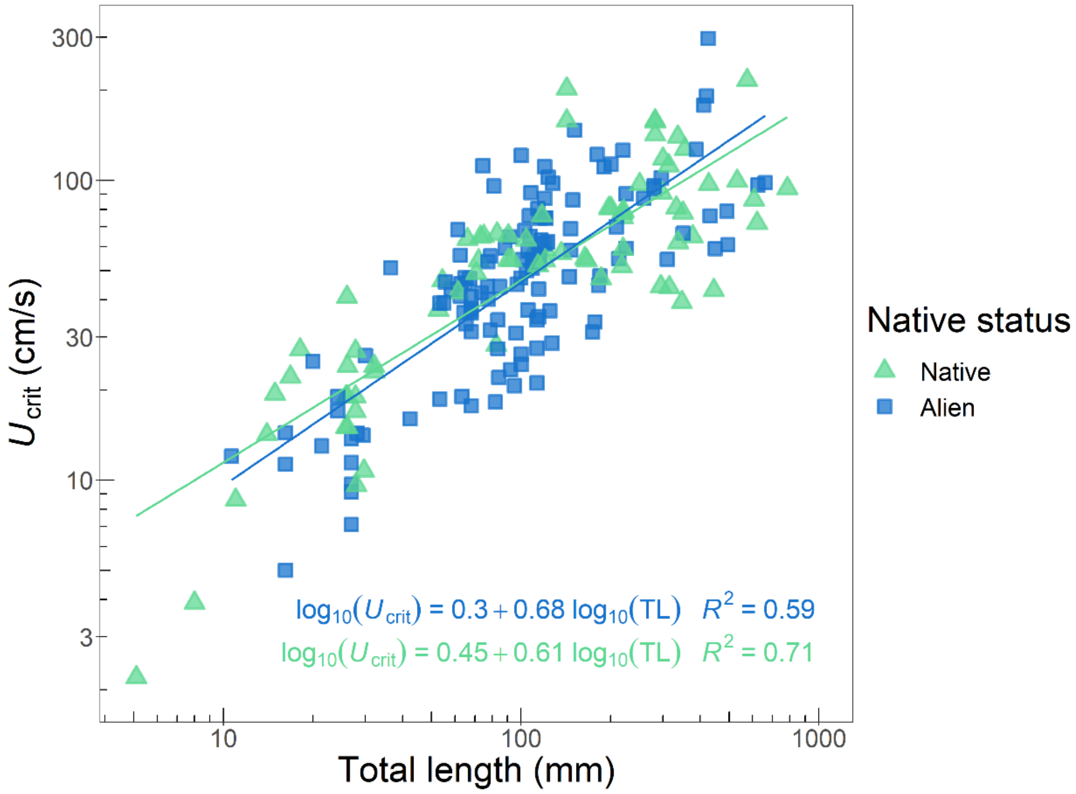

Fig. S5. Relationship of U_{crit} with fish total length (TL) (note log scales) by native status. Regression linear functions are also shown.

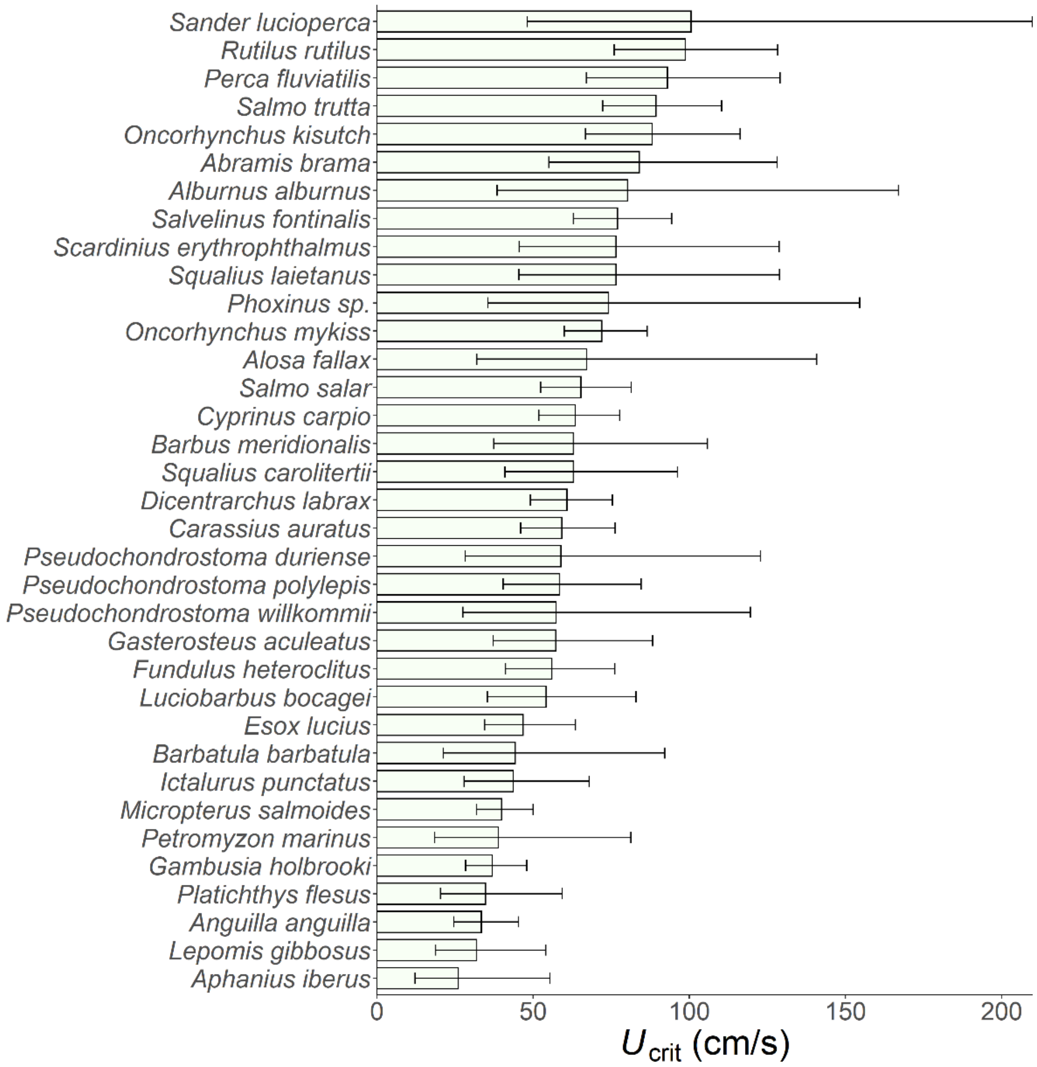

Fig. S6. Estimated marginal means (EMMs) of the ANCOVA model $\log_{10}(U_{crit})$~ $\log_{10}$(TL) + Species + Time step + Temperature + Temperature^2. Error bars represent the 95% confidence interval.

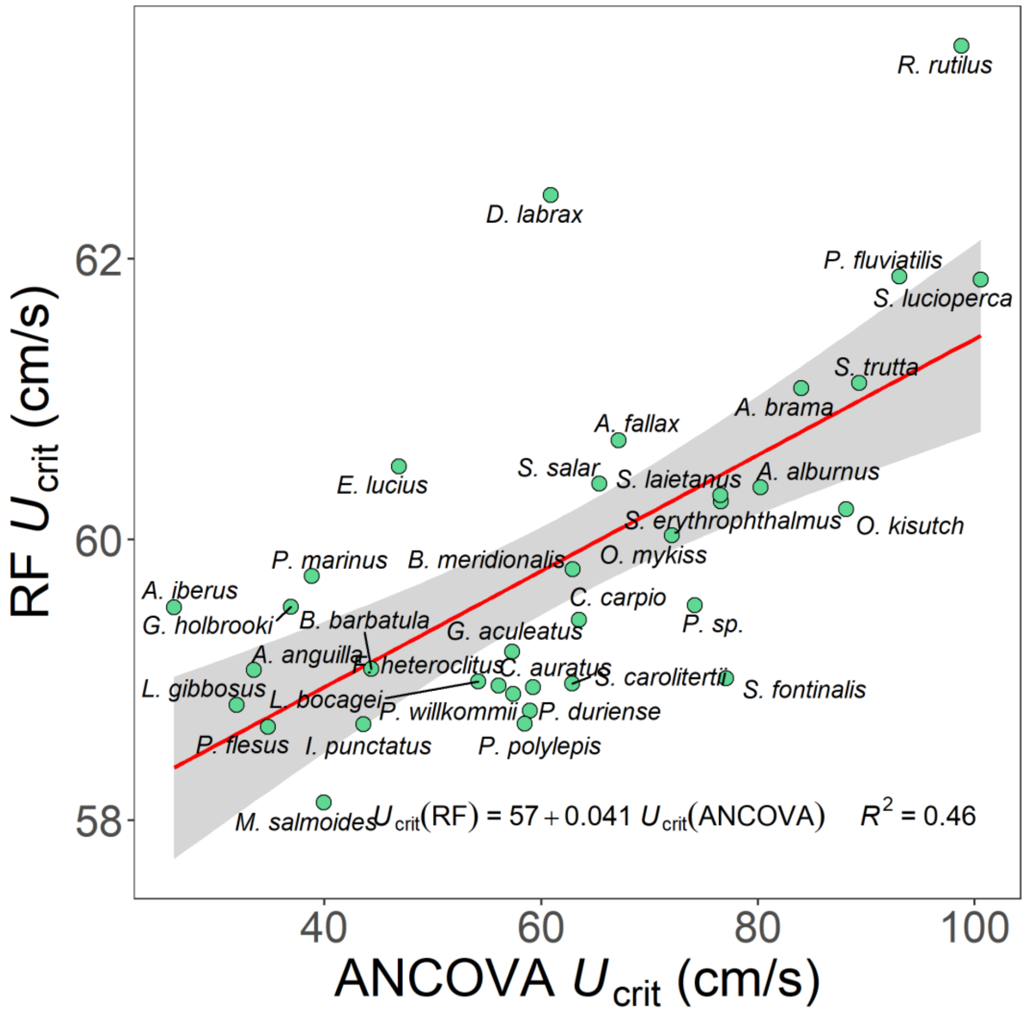

Fig. S7. Relationship of estimated U_{crit} with random forest (RF, Fig. 2) with estimated U_{crit} from ANCOVA (Fig. S5). The red line corresponds to the linear regression function and shaded areas show standard errors. Regression statistics are also given.

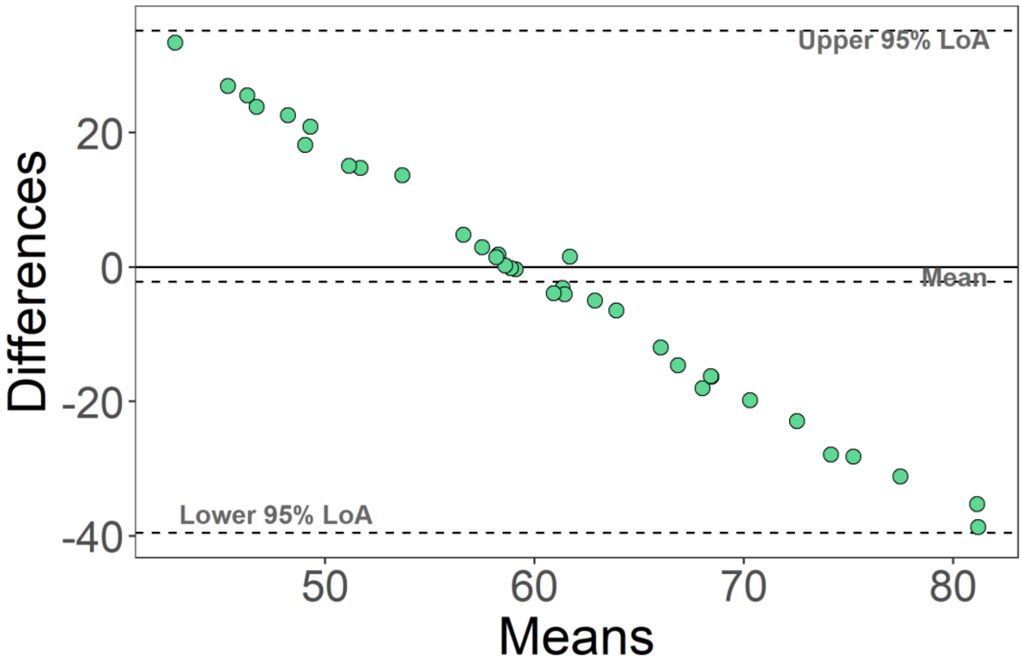

Fig. S8. Bland-Altman plot for the agreement between estimated U_{crit} with ANCOVA and random forests (RF). The solid line represents the average difference between estimated U_{crit} with the two techniques (i.e. bias), while dotted lines represent the upper and lower 95% confidence limits of agreement (LoA).

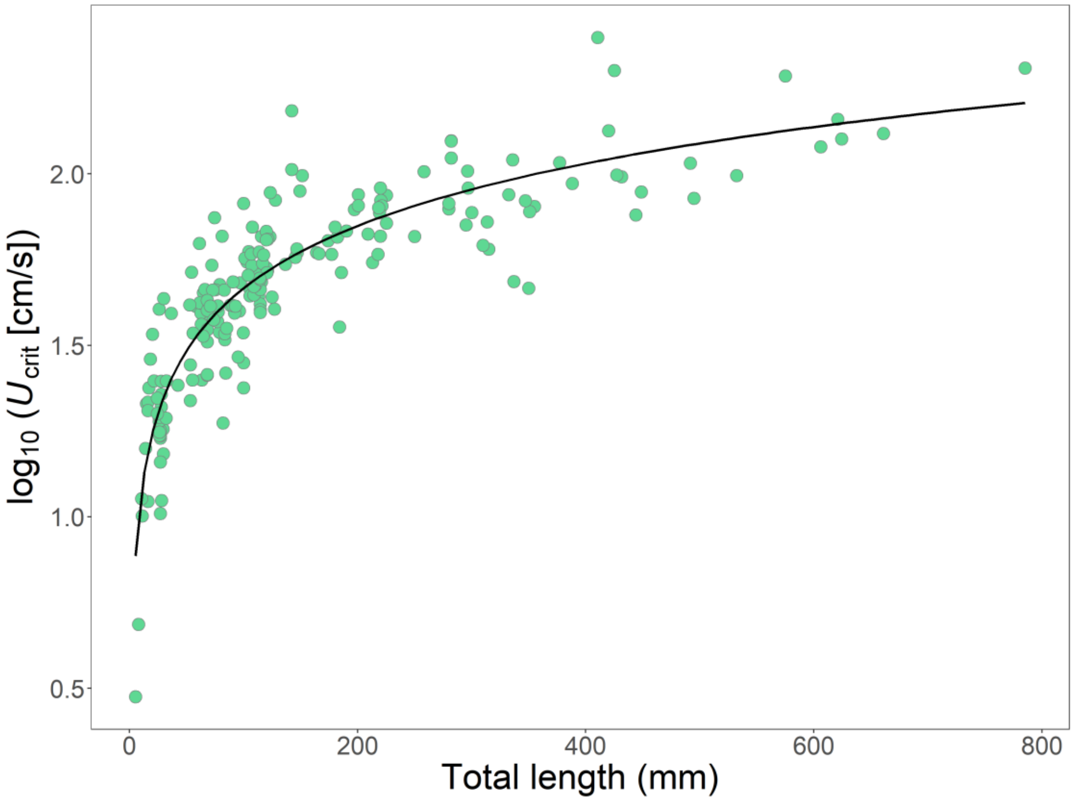

Fig. S9. Relationship of U_{crit} with fish total length (TL). The black line corresponds to the estimated LMM function. Note that U_{crit} was log_{10}-transformed.

Table S4. Summary table for all Iberian fish species (including alien species). Mean U_{crit} values and statistics for total length (TL), time step (TS) and temperature (T) and references are given. *n* = number of U_{crit} data used.

Species	U_{crit} (cm/s)	TL_{mean} (mm)	TL_{min} (mm)	TL_{max} (mm)	TS_{mean} (min)	TS_{min} (min)	TS_{max} (min)	T_{mean} (ºC)	T_{min} (ºC)	T_{max} (ºC)	*n*	References
Abramis brama	79.6	159.7	79.0	280.0	20.1	0.3	30.0	16.1	12.3	18.0	3	[1,2]
Alburnus alburnus	68.0	103.0	103.0	103.0	20.0	20.0	20.0	20.0	20.0	20.0	1	[3]
Alosa fallax	139.8	336.0	336.0	336.0	0.3	0.3	0.3	18.7	18.7	18.7	1	[4]
Anguilla anguilla	62.0	435.7	72.0	785.4	20.0	0.2	40.0	17.6	12.0	23.0	6	[5–8]
Aphanius iberus	10.8	29.6	29.6	29.6	20.0	20.0	20.0	25.0	25.0	25.0	1	[9]
Barbatula barbatula	28.2	82.6	82.6	82.6	20.0	20.0	20.0	15.0	15.0	15.0	1	[10]
Barbus meridionalis	58.2	115.5	110.0	120.9	20.0	20.0	20.0	20.5	20.0	21.0	2	[3]
Carassius auratus	39.5	75.9	64.8	97.0	19.4	15.0	20.0	18.4	10.0	25.0	9	[11–15]
Cyprinus carpio	57.7	151.0	58.3	495.0	25.7	20.0	60.0	18.2	10.0	25.0	14	[10,11,14,16–18]
Dicentrarchus labrax	52.6	99.5	5.1	355.0	7.1	2.0	20.0	20.5	14.0	25.0	17	[19–23]
Esox lucius	82.3	170.8	21.4	425.0	8.8	3.0	30.0	17.3	16.0	18.0	6	[1,24,25]
Fundulus heteroclitus	36.6	64.8	36.5	73.6	11.2	10.0	20.0	20.7	5.2	32.4	8	[26–28]
Gambusia holbrooki	13.4	24.2	16.2	30.0	12.1	5.0	20.0	20.1	10.0	30.0	12	[3,12,29–32]
Gasterosteus aculeatus	30.9	45.1	28.0	54.4	15.0	5.0	20.0	16.0	15.0	18.0	3	[33,34]
Ictalurus punctatus	57.5	195.1	147.0	225.2	26.7	20.0	30.0	24.0	20.0	27.0	3	[35–37]
Lepomis gibbosus	24.7	111.0	95.1	127.0	40.0	20.0	60.0	20.0	20.0	20.0	2	[3,38]
Luciobarbus bocagei	71.7	257.5	219.8	332.5	30.0	30.0	30.0	16.8	16.0	18.5	3	[39,40]
Micropterus salmoides	34.7	129.2	92.2	182.3	22.1	15.0	30.0	16.8	5.0	25.0	12	[41–44]
Oncorhynchus kisutch	57.9	187.6	53.3	661.1	45.9	15.0	60.0	12.3	3.0	23.0	11	[45–51]
Oncorhynchus mykiss	75.0	215.3	55.0	491.8	17.8	1.0	40.0	13.1	6.0	18.0	17	[12,24,52–60]
Perca fluviatilis	97.7	163.7	100.0	220.0	23.0	15.0	30.0	16.4	15.0	18.0	5	[1,10,12]
Petromyzon marinus	86.2	606.0	606.0	606.0	30.0	30.0	30.0	15.0	15.0	15.0	1	[61]
Phoxinus sp.	49.0	70.7	70.7	70.7	20.0	20.0	20.0	20.0	20.0	20.0	1	[3]

Species	U_{crit} (cm/s)	TL_{mean} (mm)	TL_{min} (mm)	TL_{max} (mm)	TS_{mean} (min)	TS_{min} (min)	TS_{max} (min)	T_{mean} (°C)	T_{min} (°C)	T_{max} (°C)	*n*	References
Platichthys flesus	41.7	321.0	295.0	347.0	30.0	30.0	30.0	10.0	5.0	15.0	2	[62]
Pseudochondrostoma duriense	55.0	164.0	164.0	164.0	30.0	30.0	30.0	15.0	15.0	15.0	1	[63]
Pseudochondrostoma polylepis	70.5	211.1	185.7	221.2	30.0	30.0	30.0	17.4	15.0	19.0	4	[63–65]
Pseudochondrostoma willkommii	54.0	166.0	166.0	166.0	30.0	30.0	30.0	15.0	15.0	15.0	1	[63]
Rutilus rutilus	84.1	111.0	10.7	190.1	17.9	0.3	30.0	16.3	13.0	20.0	8	[1,3,10,66,67]
Salmo salar	67.8	215.1	25.8	575.0	16.7	5.0	30.0	12.5	6.0	18.0	12	[68–73]
Salmo trutta	82.4	148.9	26.1	350.0	11.4	0.3	20.0	11.5	1.7	19.0	15	[10,57,67,69,74,75]
Salvelinus fontinalis	55.6	100.9	24.2	127.8	24.1	0.2	75.0	13.9	6.0	15.0	14	[69,76,77]
Sander lucioperca	191.0	420.0	420.0	420.0	30.0	30.0	30.0	18.0	18.0	18.0	1	[1]
Scardinius erythrophthalmus	84.5	200.0	120.0	280.0	30.0	30.0	30.0	18.0	18.0	18.0	2	[1,78]
Squalius carolitertii	54.3	123.6	114.0	136.5	30.0	30.0	30.0	18.9	18.9	19.0	3	[65,79]
Squalius laietanus	69.8	110.8	104.1	117.5	20.0	20.0	20.0	20.9	20.0	21.9	2	[3]

Supplementary references

1. Ohlmer, W. & Schwartzkopff, J. Schwimmgeschwindigkeiten von Fischen aus stehenden Binnengewässern. *Naturwissenschaften* **46**, 362–363 (1959).

2. Clough, S. C., Lee-Elliott, I. E., Turnpenny, A. W. H., Holden, S. D. J. & Hinks, C. *Swimming Speeds in Fish : phase 2 R&D Technical Report W2-049/TR1*. (2004).

3. Rubio-Gracia, F., García-Berthou, E., Guasch, H., Zamora, L. & Vila-Gispert, A. Size-related effects and the influence of metabolic traits and morphology on swimming performance in fish. *Curr. Zool.* 1–11 (2020).

4. Clough, S. C., Lee-Elliott, I. E., Turnpenny, A. W. H., Holden, S. D. J. & Hinks, C. *The swimming speeds of twaite shad (*Alosa fallax*) R&D Technical Report W2-049/TR3*. (2004).

5. McCleave, J. D. Swimming performance of European eel (*Anguilla anguilla* (L.)) elvers. *J. Fish Biol.* **16**, 445–452 (1980).

6. Mckenzie, D. J. *et al.* Tolerance of chronic hypercapnia by the European eel *Anguilla anguilla*. *J. Exp. Biol.* **206**, 1717–1726 (2003).

7. Quintella, B. B. R., Mateus, C. S., Costa, J. L., Domingos, I. & Almeida, P. R. Critical swimming speed of yellow- and silver-phase European eel (*Anguilla anguilla*, L.). *J. Appl. Ichthyol.* **26**, 432–435 (2010).

8. Tudorache, C., Burgerhout, E., Brittjin, S. & van den Thillart, G. Comparison of swimming capacity and energetics of migratory European eel (*Anguilla anguilla*) and New Zealand short-finned eel (*A. australis*). *Front. Physiol.* **6**, 1–7 (2015).

9. Rubio-Gracia, F. *et al.* Differences in swimming performance and energetic costs between an endangered native toothcarp (*Aphanius iberus*) and an invasive mosquitofish (*Gambusia holbrooki*). *Ecol. Freshw. Fish* **29**, 230–240 (2020).

10. Tudorache, C., Viaene, P., Blust, R., Vereecken, H. & De Boeck, G. A comparison of swimming capacity and energy use in seven European freshwater fish species. *Ecol. Freshw. Fish* **17**, 284–291 (2008).

11. Yan, G.-J., He, X.-K., Cao, Z.-D. & Fu, S.-J. The trade-off between steady and unsteady swimming performance in six cyprinids at two temperatures. *J. Therm. Biol.* **37**, 424–431 (2012).

12. Starrs, T., Starrs, D., Lintermans, M. & Fulton, C. J. Assessing upstream invasion risk in alien freshwater fishes based on intrinsic variations in swimming speed performance. *Ecol. Freshw. Fish* **26**, 75–86 (2015).

13. Penghan, L., Cao, Z. & Fu, S. Effect of temperature and dissolved oxygen on swimming performance in crucian carp. *Aquat. Biol.* **21**, 57–65 (2014).

14. Pang, X., Cao, Z.-D. & Fu, S.-J. The effects of temperature on metabolic interaction between digestion and locomotion in juveniles of three cyprinid fish (*Carassius auratus*, *Cyprinus carpio* and *Spinibarbus sinensis*). *Comp. Biochem. Physiol. Part A Mol. Integr. Physiol.* **159**, 253–260 (2011).

15. Yang, H., Cao, Z.-D. & Fu, S.-J. Swimming performance and energy metabolism of male and female crucian carps (*Carassius auratus*) during their reproduction phase. *Chinese J. Ecol.* **31**, 2606–2612 (2012).

16. Heap, S. P. & Goldspink, G. Alterations to the swimming performance of carp, *Cyprinus carpio*, as a result of temperature acclimation. *J. Fish Biol.* **29**, 747–753 (1986).

17. Tudorache, C., Viaenen, P., Blust, R. & De Boeck, G. Longer flumes increase critical swimming speeds by increasing burst-glide swimming duration in carp *Cyprinus carpio*, L. *J. Fish Biol.* **71**, 1630–1638 (2007).

18. West, T. G., Brauner, C. J. & Hochachka, P. W. Muscle glucose utilization during sustained swimming in the carp (*Cyprinus carpio*). *Am. J. Physiol. Integr. Comp. Physiol.* **267**, 1226–1234 (1994).

19. Carbonara, P. *et al.* Swimming performance as a well-being indicator of reared sea-bass (*Dicentrarchus labrax*). Preliminary results. *Biol. Mar. Mediterr.* **13**, 488–491 (2006).

20. Carbonara, P. *et al.* The effects of stress induced by cortisol administration on the repeatability of swimming performance tests in the European sea bass (*Dicentrarchus labrax* L.). *Mar. Freshw. Behav. Physiol.* **43**, 283–296 (2010).

21. Leis, J. M., Balma, P., Ricoux, R. & Galzin, R. Ontogeny of swimming ability in the European Sea Bass, *Dicentrarchus labrax* (L.) (Teleostei: Moronidae). *Mar. Biol. Res.* **8**, 265–272 (2012).

22. Basaran, F., Ozbilgin, H. & Ozbilgin, Y. D. Effect of lordosis on the swimming performance of juvenile sea bass (*Dicentrarchus labrax* L.). *Aquac. Res.* **38**, 870–876 (2007).

23. Basaran, F., Ozbilgin, H., Ozbilgin, Y. D., Parug, S. S. & Ozden, O. The effect of lordosis severity on juvenile sea bass (*Dicentrarchus labrax* L., 1758) swimming performance. *Turkish J. Zool.* **33**, 413–419 (2009).

24. Jones, D. R., Kiceniuk, J. W. & Bamford, O. S. Evaluation of the Swimming Performance of Several Fish Species from the Mackenzie River. *J. Fish. Res. Board Canada* **31**, 1641–1647 (1974).

25. Peake, S. Effect of Approach Velocity on Impingement of Juvenile Northern Pike at Water Intake Screens. *North Am. J. Fish. Manag.* **24**, 390–396 (2004).

26. Yetsko, K. & Sancho, G. The effects of salinity on swimming performance of two estuarine fishes, *Fundulus heteroclitus* and *Fundulus majalis*. *J. Fish Biol.* **86**, 827–833 (2015).

27. Brown, D. R., Thompson, J., Chernick, M., Hinton, D. E. & Di Giulio, R. T. Later life swimming performance and persistent heart damage following subteratogenic PAH mixture exposure in the Atlantic killifish (*Fundulus heteroclitus*). *Environ. Toxicol. Chem.* **36**, 3246–3253 (2017).

28. Fangue, N. A., Mandic, M., Richards, J. G. & Schulte, P. M. Swimming performance and energetics as a function of temperature in killifish *Fundulus heteroclitus*. *Physiol. Biochem. Zool.* **81**, 389–401 (2008).

29. Srean, P., Almeida, D., Rubio-Gracia, F., Luo, Y. & García-Berthou, E. Effects of size and sex on swimming performance and metabolism of invasive mosquitofish *Gambusia holbrooki*. *Ecol. Freshw. Fish* **26**, 424–433 (2016).

30. Seebacher, F. *et al.* Capacity for thermal acclimation differs between populations and phylogenetic lineages within a species. *Funct. Ecol.* **26**, 1418–1428 (2012).

31. Sinclair, E. L. E., De Souza, C. R. N., Ward, A. J. W. & Seebacher, F. Exercise changes behaviour. *Funct. Ecol.* **28**, 652–659 (2014).

32. Grigaltchik, V. S., Ward, A. J. W. & Seebacher, F. Thermal acclimation of interactions: Differential responses to temperature change alter predator-prey relationship. *Proc. R. Soc. B Biol. Sci.* **279**, 4058–4064 (2012).

33. Tudorache, C., Blust, R. & De Boeck, G. Swimming capacity and energetics of migrating and non-migrating morphs of three-spined stickleback *Gasterosteus aculeatus* L. and their ecological implications. *J. Fish Biol.* **71**, 1448–1456 (2007).

34. Seebacher, F., Webster, M. M., James, R. S., Tallis, J. & Ward, A. J. W. Morphological differences between habitats are associated with physiological and behavioural trade-offs in stickleback (*Gasterosteus aculeatus*). *R. Soc. Open Sci.* **3**, (2016).

35. Hocutt, C. H. Swimming Performance of Three Warmwater Fishes Exposed to a Rapid Temperature Change. *Chesap. Sci.* **14**, 11–16 (1973).

36. Beecham, R. V. *A study of the swimming capabilities of Blue,* Ictalurus furcatus*, and Channel,* I. punctatus*, catfish*. (2004).

37. Beecham, R., Thomas, T., Gao, D. X. & Gaunt, P. S. The effects of a sublethal dose of botulinum serotype E on the swimming performance of channel catfish fingerlings. *J. Aquat. Anim. Health* **26**, 149–153 (2014).

38. Brett, J. R. & Sutherland, D. B. Respiratory Metabolisrn of Purnphinseed (*Lepornis gibbosus*) in Relation to Swirnrning Speedt. *Fish. Res. Board Canada* **22**, 405–409 (1965).

39. Alexandre, C. M., Quintella, B. R., Ferreira, A. F., Romão, F. A. & Almeida, P. R. Swimming performance and ecomorphology of the Iberian barbel *Luciobarbus bocagei* (Steindachner, 1864) on permanent and temporary rivers. *Ecol. Freshw. Fish* **23**, 244–258 (2014).

40. Mateus, C. S., Quintella, B. R. & Almeida, P. R. The critical swimming speed of Iberian barbel *Barbus bocagei* in relation to size and sex. *J. Fish Biol.* **73**, 1783–1789 (2008).

41. Kolok, A. S. The Swimming Performances of Individual Largemouth Bass (*Micropterus Salmoides*) Are Repeatable. *J. Exp. Biol.* **170**, 265–270 (1992).

42. Farlinger, S. & Beamish, F. W. H. Effects of Time and Velocity Increments on the Critical Swimming Speed of Largemouth Bass (*Micropterus salmoides*). *Trans. Am. Fish. Soc.* **106**, 436–439 (1977).

43. Kolok, A. S. Photoperiod Alters the Critical Swimming Speed of Juvenile Largemouth Bass, *Micropterus salmoides*, Acclimated to Cold Water. *Copeia* **1991**, 1085–1090 (1991).

44. Cooke, S. J., Kassler, T. W. & Philipp, D. P. Physiological performance of largemouth bass related to local adaptation and interstock hybridization: Implications for conservation and management. *J. Fish Biol.* **59**, 248–268 (2001).

45. Howard, T. E. Swimming Performance of Juvenile Coho Salmon (*Oncorhynchus kisutch*) Exposed to Bleached Kraft Pulpmill Effluent. *J. Fish. Res. Board Canada* **32**, 789–793 (1975).

46. Glova, G. J. & McInerney, J. E. Critical Speeds of Coho Salmon (*Oncorhynchus kisutch*) Fry to Smolt Stages in Relation to Salinity and Temperature. *J. Fish. Res. Board Canada* **34**, 151–154 (1977).

47. Taylor, E. B. & McPhail, J. D. Variation in Burst and Prolonged Swimming Performance Among British Columbia Populations of Coho Salmon, *Oncorhynchus kisutch*. *Can. J. Fish. Aquat. Sci.* **42**, 2029–2033 (1985).

48. Griffiths, J. S. & Alderdice, D. F. Effects of Acclimation and Acute Temperature Experience on the Swimming Speed of Juvenile Coho Salmon. *J. Fish. Res. Board Canada* **29**, 251–264 (1972).

49. Brauner, C. J., Shrimpton, M. & Randall, D. Effect of Short-Duration Seawater Exposure on Plasma Ion Concentrations and Swimming Performance in Coho Salmon (*Oncorhynchus kisutch*) Parr. *Can. J. Fish. Aquat. Sci.* **49**, 2399–2405 (1992).

50. Lee, C. G. *et al.* The effect of temperature on swimming performance and oxygen consumption in adult sockeye (*Oncorhynchus nerka*) and coho (*O. kisutch*) salmon stocks. *J. Exp. Biol.* **206**, 3239–3251 (2003).

51. MacKinnon, D. L. & Farrell, A. P. The effect of 2-(Thiocyanomethylthio)Benzothiazole on juvenile Coho Salmon (*Oncorhynchus kisutch*): sublethal toxicity testing. *Exp. Toxicol. Chem.* **11**, 1541–1548 (1992).

52. Nikl, D. L. & Farrell, A. P. Reduced swimming performance and gill structural changes in juvenile salmonids exposed to 2-(thiocyanomethylthio)benzothiazole. *Aquat. Toxicol.* **27**, 245–263 (1993).

53. Fry, F. E. J. & Cox, E. T. A Relation of Size to Swimming Speed in Rainbow Trout. *J. Fish. Res. Board Canada* **27**, 976–979 (1970).

54. Hawkins, D. K. & Quinn, T. P. Critical swimming velocity and associated morphology of juvenile coastal cutthroat trout (*Oncorhynchus clarki clarki*), steelhead trout (*Oncorhynchus mykiss*), and their hybrids. *Can. J. Fish. Aquat. Sci.* **53**, 1487–1496 (1996).

55. Shingles, A. *et al.* Effects of sublethal ammonia exposure on swimming performance in rainbow trout (*Oncorhynchus mykiss*). *J. Exp. Biol.* **204**, 2691–8 (2001).

56. Gregory, T. R. & Wood, C. M. Individual variation and interrelationships between swimming performance, growth rate, and feeding in juvenile rainbow trout (*Oncorhynchus mykiss*). *Can. J. Fish. Aquat. Sci.* **55**, 1583–1590 (1998).

57. Ralph, A. L., Berli, B. I., Burkhardt-Holm, P. & Tierney, K. B. Variability in swimming performance and underlying physiology in rainbow trout (*Oncorhynchus mykiss*) and brown trout (*Salmo trutta*). *Comp. Biochem. Physiol. - A Mol. Integr. Physiol.* **163**, 350–356 (2012).

58. Jain, K. E. & Farrell, A. P. Influence of seasonal temperature on the repeat swimming performance of rainbow trout *Oncorhynchus mykiss*. *J. Exp. Biol.* **206**, 3569–3579 (2003).

59. Peake, S., McKinley, R. S. & Scruton, D. A. Swimming performance of various freshwater Newfoundland salmonids relative to habitat selection and fishway design. *J. Fish Biol.* **51**, 710–723 (1997).

60. Duthie, B. Y. G. G. & Hughes, G. M. The Effects of Reduced Gill Area and Hyperoxia on the Oxygen Consumption and Swimming Speed of Rainbow Trout. *J. Exp. Biol.* **127**, 349–354 (1987).

61. Mesa, M. G., Bayer, J. M. & Seelye, J. G. Swimming Performance and Physiological Responses to Exhaustive Exercise in Radio-Tagged and Untagged Pacific Lampreys. *Trans. Am. Fish. Soc.* **132**, 483–492 (2003).

62. Duthie, G. G. The respiratory metabolism of temperature-adapted flatfish at rest and during swimming activity and the use of anaerobic metabolism at moderate swimming speeds. *J. Exp. Biol.* **97**, 359–373 (1982).

63. Branca, R. X. M. *Capacidade natatória e ecomorfologia de três espécies de bogas (*Pseudochondrostoma polylepis*,* Pseudochondrostoma duriense*,* Pseudochondrostoma willkommii*) em rios portugueses*. (2015).

64. Alexandre, C. M., Branca, R., Quintella, B. R. & Almeida, P. R. Critical swimming speed of the southern straight-mouth nase *Pseudochondrostoma willkommii* (Steindachner, 1866), a potamodromous cyprinid from southern Europe. *Limnetica* **35**, 365–372 (2016).

65. Romão, F., Quintella, B. R., Pereira, T. J. & Almeida, P. R. Swimming performance of two Iberian cyprinids: The Tagus nase *Pseudochondrostoma polylepis* (Steindachner, 1864)

and the bordallo *Squalius carolitertii* (Doadrio, 1988). *J. Appl. Ichthyol.* **28**, 26–30 (2012).

66. Mann, R. H. K. & Bass, J. A. B. The critical water velocities of larval roach (*Rutilus rutilus*) and dace (*Leuciscus leuciscus*) and implications for river management. *Regul. Rivers Res. Manag.* **13**, 295–301 (1997).

67. Clough, S. C. & Turnpenny, A. W. H. *Swimming speeds in fish: Phase 1 R&D Technical Report W2-026/TR1*. (2001).

68. Booth, R. K., Scott McKinley, R., Økland, F. & Sisak, M. M. In situ measurement of swimming performance of wild Atlantic salmon (*Salmo salar*) using radio transmitted electromyogram signals. *Aquat. Living Resour.* **10**, 213–219 (1997).

69. Heggenes, J. & Traaen, T. Downstream migration and critical water velocities in stream channels for fry of four salmonid species. *J. Fish Biol.* **32**, 717–727 (1988).

70. Hvas, M. & Oppedal, F. Sustained swimming capacity of Atlantic salmon. *Aquac. Environ. Interact.* **9**, 361–369 (2017).

71. Bui, S., Dempster, T., Remen, M. & Oppedal, F. Effect of ectoparasite infestation density and life-history stages on the swimming performance of Atlantic salmon *Salmo salar*. *Aquac. Environ. Interact.* **8**, 387–395 (2016).

72. Remen, M. *et al.* Critical swimming speed in groups of Atlantic salmon *Salmo salar*. *Aquac. Environ. Interact.* **8**, 659–664 (2016).

73. McCleave, J. D. & Stred, K. A. Effect of Dummy Telemetry Transmitters on Stamina of Atlantic Salmon (*Salmo salar*) Smolts. *J. Fish. Res. Board Canada* **32**, 559–563 (1975).

74. Butler, P. J., Day, N. & Namba, K. Interactive effects of seasonal temperature and low pH on resting oxygen uptake and swimming performance of adult brown trout *Salmo trutta*. *J. Exp. Biol.* **165**, 195–212 (1992).

75. Taugbøl, A., Olstad, K., Bærum, K. M. & Museth, J. Swimming performance of brown trout and grayling show species-specific responses to changes in temperature. *Ecol. Freshw. Fish* **28**, 241–246 (2019).

76. Peterson, R. H. Influence of Fenitrothion on Swimming Velocities of Brook Trout (*Salvelinus fontinalis*). *J. Fish. Res. Board Canada* **31**, 1757–1762 (1974).

77. Beamish, F. W. H. Swimming performance and oxygen consumption of the charrs. in *Charrs. Salmonid Fishes of the Genus Salvelinus* (ed. Balon, E. K.) 739–748 (1980).

78. Pavlov, D. S., Sbikin, Y. N., Vashchinnikov, A. E. & Mochek, A. D. The effect of light intensity and water temperature on current velocities critical to fish. *Vopr. Ikhtiologii* **12**, 769–778 (1972).

79. Romão, F. A. S. *Determinação das velocidades críticas de natação da boga-comum (*Pseudochondrostoma polylepis *Steindachner, 1865) e do escalo do Norte (*Squalius carolitertii *Doadrio, 1988)*. (2009).
